# Using Molecular Characterization to Support Investigations of Aquatic Facility–Associated Outbreaks of Cryptosporidiosis — Alabama, Arizona, and Ohio, 2016

**DOI:** 10.15585/mmwr.mm6619a2

**Published:** 2017-05-19

**Authors:** Michele C. Hlavsa, Dawn M. Roellig, Matthew H. Seabolt, Amy M. Kahler, Jennifer L. Murphy, Taishayla K. McKitt, Evelyn F. Geeter, Ron Dawsey, Sherri L. Davidson, Thuy N. Kim, Theresa H. Tucker, Sally Ann Iverson, Brenna Garrett, Nicole Fowle, Jennifer Collins, Gregory Epperson, Scott Zusy, Joli R. Weiss, Ken Komatsu, Edwin Rodriguez, J. Gage Patterson, Rebecca Sunenshine, Brandi Taylor, Katie Cibulskas, Lynn Denny, Keoni Omura, Boris Tsorin, Kathleen E. Fullerton, Lihua Xiao

**Affiliations:** ^1^Division of Foodborne, Waterborne, and Environmental Diseases, National Center for Emerging and Zoonotic Infectious Diseases, CDC; ^2^Alabama Department of Public Health; ^3^Maricopa County Department of Public Health, Arizona; ^4^Arizona Department of Health Services; ^5^Epidemic Intelligence Service, CDC; ^6^Maricopa County Environmental Services Department, Arizona; ^7^Coconino County Public Health Services District, Arizona; ^8^Arizona State Public Health Laboratory; ^9^Career Epidemiology Field Officer Program, CDC; ^10^Ohio Department of Health; ^11^Ohio Department of Health Laboratory.

Cryptosporidiosis is a nationally notifiable gastrointestinal illness caused by parasitic protozoa of the genus *Cryptosporidium*, which can cause profuse, watery diarrhea that can last up to 2–3 weeks in immunocompetent patients and can lead to life-threatening wasting and malabsorption in immunocompromised patients. Fecal-oral transmission of *Cryptosporidium* oocysts, the parasite’s infectious life stage, occurs via ingestion of contaminated recreational water, drinking water, or food, or following contact with infected persons or animals, particularly preweaned bovine calves (*1*). The typical incubation period is 2–10 days. Since 2004, the annual incidence of nationally notified cryptosporidiosis has risen approximately threefold in the United States (*1*). *Cryptosporidium* also has emerged as the leading etiology of nationally notified recreational water–associated outbreaks, particularly those associated with aquatic facilities (i.e., physical places that contain one or more aquatic venues [e.g., pools] and support infrastructure) (*2*). As of February 24, 2017, a total of 13 (54%) of 24 states reporting provisional data detected at least 32 aquatic facility–associated cryptosporidiosis outbreaks in 2016. In comparison, 20 such outbreaks were voluntarily reported to CDC via the National Outbreak Reporting System for 2011, 16 for 2012, 13 for 2013, and 16 for 2014. This report highlights cryptosporidiosis outbreaks associated with aquatic facilities in three states (Alabama, Arizona, and Ohio) in 2016. This report also illustrates the use of CryptoNet, the first U.S. molecularly based surveillance system for a parasitic disease, to further elucidate *Cryptosporidium* chains of transmission and cryptosporidiosis epidemiology. CryptoNet data can be used to optimize evidence-based prevention strategies. Not swimming when ill with diarrhea is key to preventing and controlling aquatic facility–associated cryptosporidiosis outbreaks (https://www.cdc.gov/healthywater/swimming/swimmers/steps-healthy-swimming.html).

## Alabama

On August 12, 2016, the Alabama Department of Public Health received a report of 35 persons who developed gastrointestinal symptoms after visiting an Alabama aquatic facility. Case-finding efforts identified 23 outbreak-associated cases. Three (13%) patients had laboratory-confirmed *Cryptosporidium* infection; molecular characterization by CryptoNet of one *Cryptosporidium* specimen identified it as the *C. hominis* IfA12G1R5 subtype. Data collected using an outbreak-specific questionnaire completed for 15 patients indicated the median incubation period was 8 days (range = 5–17 days) after visiting the aquatic facility on July 31. The limited number of completed questionnaires provided insufficient statistical power to determine outbreak risk factors. On August 15, investigators collected filter backwash and water samples directly from the facility’s aquatic venues; on August 16, facility operators hyperchlorinated the aquatic venues, raising the free available chlorine concentration for a prolonged period to achieve 3-log_10_ (99.9%) *Cryptosporidium* inactivation. Microscopy and real-time polymerase chain reaction (PCR) testing (*3*) did not detect *Cryptosporidium* in the filter backwash or water samples. An inspection found facility operation and maintenance in compliance with local standards, including water disinfectant concentration and pH standards. No fecal incident was known to have occurred in any of the facility’s aquatic venues before the outbreak. However, it was recommended the facility develop policies on fecal incident response and maintain a fecal/vomit incident response log.*

## Arizona

On August 2, 2016, the Arizona Department of Health Services was notified of a cluster of gastrointestinal illness among players on a Coconino County Little League team and family members; 36 (71%) of 51 persons became ill 6–7 days after visiting a Maricopa County aquatic facility on July 22. Molecular characterization by CryptoNet of four *Cryptosporidium* specimens from the Little League cohort identified all four as the *C. hominis* IfA12G1R5 subtype. Maricopa County Department of Public Health simultaneously detected increased laboratory reporting of cryptosporidiosis as of mid-July. Multiple patients reported visits to the same Maricopa County aquatic facility. To determine the magnitude of the outbreak, the counties interviewed cryptosporidiosis patients, focusing on possible risk factors, particularly recreational water exposures. During July 1–October 31, 2016, a total of 352 laboratory-confirmed cryptosporidiosis cases were detected statewide, compared with a median of 46 total cases (range = 42–62) detected annually during 2011–2015. Among 317 interviewed patients, 204 (64%) reported recreational water exposure at 86 public aquatic venues, 74 (86%) of which were in Maricopa County. Environmental health practitioners of affected counties worked with facility operators to hyperchlorinate identified aquatic venues. Among 247 Maricopa County patients interviewed, 43 (17%) reported swimming while symptomatic at a median of one venue (range = 1–3).

## Ohio

During 2012–2015, the Ohio Department of Health and local public health partners detected a median of 399 cryptosporidiosis cases annually statewide (range = 324–571). In 2016, annual incidence increased nearly fivefold to 1,940 cases. Ten (42%) of 24 cryptosporidiosis outbreaks detected in Ohio in 2016^†^ were associated with aquatic venues. Assessing patients’ recreational water exposures to determine the magnitude of individual recreational water–associated outbreaks was complicated by individual patients reporting multiple exposures during their incubation period. Among six *Cryptosporidium* specimens from patients affected by these outbreaks, all were identified by CryptoNet as the *C. hominis* IdA19 subtype, which has rarely been identified in the United States. Five specimens were from a university sports team’s members; the sixth specimen was from a patient with no epidemiologic link to the university sports team except visiting the same waterpark. The matching subtype and epidemiologic link led the Ohio Department of Health to classify the 26 cases in the university sports team as part of the waterpark–associated outbreak; these cases previously had been thought to be associated with a different outbreak.

## Discussion

This report highlights cryptosporidiosis outbreaks associated with aquatic facilities in three states in 2016. CryptoNet genotyping (18S PCR-RFLP) to determine *Cryptosporidium *species and subtyping (gp60 PCR and sequencing) to determine subtype (*4*) supported and strengthened the Alabama, Arizona, and Ohio outbreak investigations. First, molecular characterization identified or confirmed epidemiologic links among individual outbreak-associated cases. Second, and perhaps more importantly, *C. hominis* was repeatedly identified as the outbreak etiology. Given that individual *Cryptosporidium* species can have unique host ranges, identifying the *Cryptosporidium* species can provide insight into possible exposures and outbreak sources. Identifying *C. hominis* as the etiology of these outbreaks indicates a human source of contamination and underscores the need to engage swimmers and parents of young swimmers in efforts to prevent and control aquatic facility–associated cryptosporidiosis outbreaks.

Most *Cryptosporidium* species are indistinguishable by traditional diagnostic tests (microscopy or immunoassays); only molecular diagnostic methods, such as those used by CryptoNet, can distinguish these species and their subtypes. *C. hominis* IfA12G1R5 subtype was identified as the etiology in the Alabama and Arizona outbreak investigations. This subtype was initially identified in small numbers of specimens from sporadic (i.e., not outbreak-associated) cryptosporidiosis cases in the United Kingdom and Australia (*5*–*7*). In the United States, it was first seen in specimens from patients with acquired immunodeficiency syndrome (AIDS) and was responsible for a 2009 Oregon cryptosporidiosis outbreak associated with the care of an AIDS patient. Since 2013, it has emerged as the dominant *C. hominis* subtype among sporadic and outbreak-associated cases with *Cryptosporidium* subtyping data; 107 (36.6%) of 292 *Cryptosporidium* specimens from sporadic cases in 2016 were identified as the *C. hominis* IfA12G1R5 subtype.

To better understand the implication of identifying this subtype, molecular characterization of *Cryptosporidium* specimens needs to shift from predominantly supporting outbreak investigations to becoming nationally systematic. In 2010, CDC launched CryptoNet (https://www.cdc.gov/parasites/crypto/cryptonet.html), the first U.S. molecularly based surveillance system for a parasitic disease. Formal collaborations with state public health partners began in mid-2015. The objectives are to efficiently integrate CryptoNet into existing infrastructure when possible (e.g., merging the CryptoNet BioNumerics infrastructure into that of PulseNet) and to regularly analyze molecular characterization and epidemiologic data for each nationally notified case of cryptosporidiosis to further elucidate *Cryptosporidium* chains of transmission and cryptosporidiosis epidemiology (e.g., geographic and temporal changes in the distribution of *Cryptosporidium* species and their subtypes and associated exposures). Achieving these objections requires overcoming barriers to successful molecular characterization and sharing epidemiologic data by 1) increasing the positive predictive value of rapid diagnostic tests (i.e., decreasing the frequency of false positive results) (*8*), 2) shifting away from fixing specimens in formalin (which precludes molecular characterization), 3) advancing molecular diagnostics from single-gene to multilocus or whole-genome sequencing (which will increase discriminatory power), and 4) increasing state capacity to collect and share epidemiologic data with CDC.

The emergence of *Cryptosporidium* as the leading etiology of aquatic facility–associated outbreaks results from the parasite’s extreme chlorine tolerance. Free available chlorine inactivates most infectious pathogens within minutes at CDC-recommended concentrations of at least 1 ppm^§^; however, *Cryptosporidium* oocysts can survive for days (*9*). As the Alabama outbreak investigation indicates, even properly operated and maintained aquatic venues can be sites of *Cryptosporidium* transmission. In addition, cyanuric acid (a stabilizer added to prevent chlorine depletion by the sun’s ultraviolet light) has been found to substantially delay chlorine inactivation of *Cryptosporidium* (*9*). Consequently, in July 2016, CDC issued revised recommendations for hyperchlorination (https://www.cdc.gov/healthywater/swimming/aquatics-professionals/fecalresponse.html) when responding to diarrheal incidents in public aquatic venues (i.e., high-risk *Cryptosporidium* contamination events) and aquatic facility–associated cryptosporidiosis outbreaks. These recommendations are also included in CDC’s 2016 Model Aquatic Health Code (https://www.cdc.gov/mahc/editions/current.html). This national guidance can be adopted voluntarily by state and local jurisdictions and aquatic facilities to minimize the risk for public aquatic facility–associated illness and injury, particularly cryptosporidiosis.

Preventing *Cryptosporidium* contamination of water in an aquatic venue would prevent *Cryptosporidium* transmission more efficiently than remediating actions once contamination occurs. This means that public health agencies and the aquatics sector need to collaborate on engaging swimmers, who are the source of contamination, in prevention efforts. Young swimmers aged <5 years are more likely to contaminate the water because they are more likely to have inadequate toileting and hygiene skills; therefore, prevention efforts should focus on their parents. As the Arizona outbreak investigation demonstrated, patients continue to swim while symptomatic. The key healthy swimming message to the public to prevent contamination is “Don't swim or let your kids swim if sick with diarrhea.” Health care providers should also instruct cryptosporidiosis patients not to go back into the water until they have been diarrhea-free for 2 weeks.^¶ ^Healthy swimming promotion campaigns conducted before the summer swim season could reduce the risk for outbreaks caused by *Cryptosporidium* and other enteric pathogens (*10*), while optimizing the health benefits of water-based physical activity (https://www.cdc.gov/healthywater/swimming/swimmers/health_benefits_water_exercise.html).

**Summary**

**What is already known about this topic?**

*Cryptosporidium* has emerged as the leading etiology of recreational water–associated outbreaks, particularly those associated with aquatic facilities (places that contain one or more aquatic venues [e.g., swimming pools, interactive water play venues or water playgrounds, or hot tubs/spas] and support infrastructure [e.g., chemical storage space]).

**What is added by this report?**

Most *Cryptosporidium* species are indistinguishable by traditional diagnostic tests (microscopy or immunoassays); only molecular diagnostic methods, such as those used by CryptoNet, the first U.S. molecularly based surveillance system for a parasitic disease, can distinguish these species and their subtypes. Given that individual *Cryptosporidium* species can have unique host ranges, identifying the *Cryptosporidium* species can provide insight into possible exposures and outbreak sources. In the summer of 2016, when detection of cryptosporidiosis outbreaks increased, CryptoNet supported outbreak investigations by further elucidating *Cryptosporidium* chains of transmission.

**What are the implications for public health practice?**

Regular analysis of molecular characterization and epidemiologic data through CryptoNet for each nationally notified cryptosporidiosis case can further elucidate *Cryptosporidium* chains of transmission and cryptosporidiosis epidemiology (e.g., by monitoring geographic and temporal changes in the distribution of *Cryptosporidium* species and their subtypes and associated exposures). CryptoNet data can then be used to optimize development of evidence-based prevention strategies. Not swimming when ill with diarrhea is key to preventing and controlling aquatic facility–associated cryptosporidiosis outbreaks (https://www.cdc.gov/healthywater/swimming/swimmers/steps-healthy-swimming.html). State and local jurisdictions and aquatic facilities can voluntarily adopt recommendations in CDC’s Model Aquatic Health Code (https://www.cdc.gov/mahc/editions/current.html) to prevent and control *Cryptosporidium* transmission in public aquatic venues.

## Footnotes

* An example of an aquatic venue water-contamination–response log is available at https://www.cdc.gov/healthywater/pdf/swimming/pools/water-contamination-response-log.pdf.

† During 2012–2015, the Ohio Department of Health and local public health partners annually detected a median of six (range = 3–7) cryptosporidiosis outbreaks, a median of one (range = 0–2) were associated with recreational water.

§ At water pH 7.2–7.8 and temperature 77°F (25°C).

¶ The additional 2 weeks are recommended only for patients whose diarrhea is known to be caused by *Cryptosporidium*, because diarrhea caused by *Cryptosporidium* can repeatedly wax and wane before complete resolution, and cryptosporidiosis patients can continue to excrete infectious *Cryptosporidium* oocysts, typically for up to 2 weeks, after symptoms completely resolve.
